# Amplification by bone conduction in congenital malformations: patient benefits and satisfaction

**DOI:** 10.5935/1808-8694.20130063

**Published:** 2015-10-04

**Authors:** Elaine Cristina Moreto Paccola, João Cândido Fernandes, Maria Fernanda Capoani Garcia Mondelli

**Affiliations:** a MSc in Rehabilitation Sciences at the Craniofacial Rehabilitation Hospital - University of São Paulo (Speech and Hearing Therapist at the Craniofacial Rehabilitation Hospital - University of São Paulo).; b Professor in the Department of Mechanical Engineering of the Julio de Mesquita Filho Paulista State University.; c PhD in Rehabilitation Sciences at the Craniofacial Rehabilitation Hospital - University of São Paulo (Professor in the Bauru Dentistry School - USP). Craniofacial Rehabilitation Hospital - University of São Paulo.

**Keywords:** bone conduction, congenital abnormalities, hearing aids, patient satisfaction, questionnaires

## Abstract

Hearing loss is one of the most common clinical findings in subjects with malformations of the ear. Treatment consists of surgery and/or adapt a hearing aid amplification by bone (HA VO). Early intervention is critical to auditory stimulation and development of speech and language.

**Objective:**

To characterize the audiological profile of subjects with congenital malformation of the external ear and/or middle and evaluate the benefit and satisfaction of using HA VO.

**Method:**

A descriptive study, subjects with bilateral congenital malformations of the external ear and/ or middle, conductive or mixed hearing loss, moderate or severe and HA VO users. Evaluation of the benefit test using sentence recognition in noise and measures of functional gain and satisfaction assessment questionnaire using international IQ - HA.

**Results:**

13 subjects were evaluated, 61% were male and 80% with moderate conductive hearing loss or severe. There was better performance in the evaluation proposal, provided with HA when compared to the condition without HA.

**Conclusion:**

HA VO showed advantages for the population studied and should be considered as an option for intervention. Satisfaction was confirmed by elevated scores obtained in IQ - HA.

## INTRODUCTION

Ear malformations occur during embryo development and may affect the outer, middle and inner ear. The more common congenital ear anomalies affect the pinna and the ear canal, involving one or both ears. Hearing loss is one of the most common findings in individuals with ear malformations and may occur in varying degrees and types, depending on the level of involvement^1^.

Treatment consists of surgery and/or fitting patients with hearing aids. Early fitting of hearing aids favors auditory stimulation and the development of speech and language acquisition. Surgery is not indicated in all cases, and is usually performed in subjects aged at least six or seven years^2^.

Conventional hearing aids cannot be offered to patients with ear agenesis or ear canal stenosis, given the impossibility of using air conduction to stimulate hearing^3^. In these cases, bone conduction hearing aids represent the best option.

Bone conduction hearing aids are designed to bypass the defective middle ear to stimulate the structures inside the cochlea. The output transducer is a bone oscillator. The vibrations of the bone oscillator must be effectively transmitted to the skull. In order to achieve proper transmission, the bone oscillator is usually mounted on one side of the head, attached to an elastic band to keep it pressed against the skull. The hearing aid may be located in the other end of the headband, or close to the user's body as in conventional hearing aids^4^.

Research indicates that the amplification provided by bone conduction hearing aids, the headaches produced by the pressure applied by the headband against the mastoid, and the adverse psychosocial impact felt by users are some of the issues associated with wearing these devices. Complaints of headache caused by the headband and dissatisfaction with the visibility of the hearing aids are common in pre-teens and teenagers. These factors combined lead to reduced usage of hearing aids^5^.

Bone-anchored hearing aids (BAHA) are an advantageous alternative to conventional bone conduction hearing aids. BAHA have been studied for over two decades and their use is well established in Europe and the USA. More than 20,000 patients have been implanted with BAHA in the world^6^.

The decision on the choice of hearing aids to be offered must be made together with the patient and his/ her family. Bone conduction hearing aids are a practical, non-invasive option available at some public health care services.

This study aims to characterize the audiological profile of individuals with congenital malformations of the ear and/or middle ear and assess the benefits and level of satisfaction achieved with bone conduction hearing aids.

## METHOD

This study was approved by the Ethics Committee for Research with Human Beings and granted permit #018/2006. Subjects were advised of the nature of the study and asked to sign an informed consent term.

The following enrollment criteria were adopted:

• Age between six and 40 years
• Bilateral congenital malformation involving the ear and/or middle ear
• Bilateral moderate to severe conductive or mixed hearing loss^7^
• Experience wearing retroauricular bone conduction hearing aids for at least three months
• No cognitive disorders.

The hearing aids worn by the individuals enrolled in this study were the Unitron UE12 PPL and the Siemens™ Phoenix 213, coupled to a metal rod, a wire, and a bone oscillator of the same brand as the device.

Speech recognition tests, functional gain measurements, and the IOI - HA questionnaire were used to assess the subjects.

### Speech recognition in noise

A two-channel audiometer was used in these tests, as dictated by the standards published by the American National Standards Institute (ANSI 1991, 1996). A CD player, a stereo amplifier, and two loudspeakers (one playing speech and the other noise) were connected to the audiometer.

The material^8^ used in the tests was made up of seven lists of sentences of everyday conversation and babble noise from the commercially available CD List of Sentences in Portuguese.

The lists comprised ten short, easily repeatable affirmative sentences ranging between four and seven words in length. The sentences in the list were similar in terms of phonetic content and structure. The lists contained 48 to 54 words and 202 to 214 characters each. The sentences were recorded by a male professional announcer.

The first sentence in each list was presented at an intensity of 65 dBA to assess speech recognition thresholds in noise (SRTN). The individuals in the sample had the hearing thresholds required to perceive speech at such intensity. Noise was played at 65 dBA, in a way to start the test with a signal to noise ratio of 0 dB, as suggested by the author. When the subjects were able to correctly recognize the spoken sentence and repeat it using the same phonologic pattern, the next sentence would be played at an intensity of 4 dB less than the previous sentence. In the event of a mistake, the signal was increased in steps of 2 dB, until the individual got it right. Steps of two decibels would then be applied upwards in the event of other mistakes and downwards when the individual was successful, until all ten sentences were played.

The subjects were positioned one meter away from the loudspeakers at an angle of incidence from the noise and speech source of zero degrees azimuth.

### Measurements of functional gain

The functional gain provided by hearing aids is defined by the difference in decibels between the free-field audiometry thresholds assessed with the subjects with and without hearing aids under equal testing conditions.

The minimum intensity was 30 dB NPS with and without the use of hearing aids, as this is the minimum level of intensity allowed by the equipment used in the test sessions.

Hearing thresholds at 6000 and 8000 Hz were not acquired, as the hearing aids worn by the subjects in this study provided less gain in these frequencies.

The limit psychometric method was used to acquire hearing thresholds. The intensity of the sound stimuli was gradually reduced in steps of 10 dB until the patients could no longer respond. Then, the intensity was increased in steps of 5 dB until the subjects were able to respond again. The threshold was defined as the intensity at which patients were able to respond to 50% of the sentences presented.

### Satisfaction evaluation

The Brazilian Portuguese version of the International Outcome Inventory for Hearing Aids (IOI - HA)^9^ was used to assess patient satisfaction.

The questionnaire contains seven questions with five possible answers each and is used to assess subject adaptation to hearing aids considering the following issues: 1 - Hearing aid daily use; 2 - Benefit; 3 - Residual activity limitations; 4 - Satisfaction; 5 - Residual participation restrictions; 6 - Impact on others; 7 - Quality of life.

The questionnaire offers five possible answers graded from left to right, in a way that the first option refers to poor performance corresponding to a score of one, and the last option reflects top performance and a score of five. Caretakers/guardians were advised along with the patients to choose only one answer to characterize how well adapted the subjects were to their hearing aids.

The analysis of the IOI - HA questionnaire was based on the answers provided to each question individually and as a group. The scores on each question, the total score considering the seven questions, and the scores considering factors 1 and 2 in the questionnaire^9^ were taken into account. Factor 1 comprised questions 1, 2, 4, and 7 and intended to describe the interaction between the user and the hearing aids. Factor 2 considered questions 3, 5, and 6 and depicted the relationship between the user and the environment.

Each of the seven questions have a minimum possible score of one and a maximum possible score of five. The total score refers to the sum of the scores given to each answer and may range from seven to 35. Scores on factor 1 may range from four to 20 points, while factor 2 can vary between three and 15 points. Higher scores indicate better outcome in regards to adaptation to hearing aids. *Student's t*-test for paired samples was used to compare measurements with and without hearing aids. *Student's t*-test was used to estimate the mean scores on the questionnaire.

## RESULTS

Thirteen individuals (eight males and five females) wearing hearing aids for a mean of three years were included in the study. Their ages ranged between six and 37 years (mean of 14 years of age) (Table 1).

**Table 1:** Distribution of subjects by gender, age, right ear hearing loss, left ear hearing loss, model of fitted hearing aids, time wearing hearing aids, and use of hearing aids.

Subject	Gender	Age (years)	REHL	LEHR	Fitted hearing aids	Time wearing hearing aids (years)	Use of hearing aids
1	F	12.9	SC	MC	UE12 PPL	2.22	Systematic
2	M	9.3	MC	MC	UE12 PPL	4.41	Not systematic
3	F	14.4	SC	SC	UE12 PPL	4.43	Systematic
4	M	37.2	SM	SC	Phoenix 203	2.05	Systematic
5	M	9.9	SM	SC	Phoenix 203	2.43	Systematic
6	M	16.8	MC	MC	UE12 PPL	2.52	Systematic
7	M	10.5	MC	CG	Phoenix 203	2.22	Systematic
8	M	21.0	SM	SM	UE12 PPL	3.01	Systematic
9	M	13.8	MM	MM	UE12 PPL	3.03	Systematic
10	F	11.3	SM	SM	Phoenix 203	3.00	Systematic
11	M	6.3	MC	SC	Phoenix 203	3.00	Systematic
12	F	8.7	MC	SC	UE12 PPL	5.08	Systematic
13	F	7.9	MC	SC	Phoenix 203	7.19	Systematic

F: Female; M: Male; REHL: Right ear hearing loss; LEHR: Left ear hearing loss; SC: Severe conductive; MC: Moderate conductive; SM: Severe mixed; MM: Moderate mixed.

Descriptive and inferential statistical analysis (95% confidence interval) of the results derived from the assessment of the 13 subjects with and without hearing aids, in tests to assess speech recognition thresholds in noise (SRTN) at 65 dB A and signal to noise ratios (SNR) are presented on Table 2.

**Table 2:** SRTN (dBA) and SNR (dB) without hearing aids (WOHA) and with hearing aids (WHA) of each subject in the sample.

Subject	SRTN WOHA (dB A)	SRTN WHA (dB A)	SNR WOHA (dB)	SNR WHA (dB)
1	69.50	65.00	+5.50	-3.20
2	68.50	61.00	+3.50	-4.00
3	67.22	57.89	+2.22	-7.11
4	70.00	65.44	+5.00	+0.44
5	64.11	56.14	-0.89	-8.86
6	74.43	61.89	+9.43	-3.11
7	66.50	58.00	+1.50	-7.00
8	75.33	66.33	+10.33	+1.33
9	73.35	67.11	+7.30	-8.50
10	72.60	55.89	+7.60	-9.11
11	68.00	59.00	+3.00	-6.00
12	73.00	71.00	+8.00	+6.00
13	75.00	60.00	+10.00	-5.00

SRTN: Speech recognition threshold in noise; SNR: Signal to noise ratio; WOHA: Without hearing aids; WHA: With hearing aids.

Comparisons between the test outcomes of the 13 individuals with and without hearing aids, in terms of free-field audiometry hearing thresholds (dB NPS) at 250 to 4000 Hz, along with the measurements of functional gain in each of the tested frequencies are presented on Table 3.

**Table 3:** Summary of the measurements of free-field auditory thresholds (dB NPS) without hearing aids, with hearing aids, and difference between without and with hearing aids for frequencies ranging from 250 to 4000 Hz.

Variables		n	Mean	Standard deviation	Minimum	Median	Maximum
	WOHA	13	56	10.6	40	60	70
250 Hz	WHA	13	36	6.1	30	35	45
	WHOA-WHA	13	21	10.2	5	25	35

	WOHA	13	59	7.3	50	60	75
500 Hz	WHA	13	32	3.8	30	30	40
	WHOA-WHA	13	27	6.3	15	25	40

	WOHA	13	56	5.1	50	55	65
1000 Hz	WHA	13	31	1.9	30	30	35
	WHOA-WHA	13	25	4.8	20	25	35

	WOHA	13	53	6.9	40	55	65
2000 Hz	WHA	13	32	2.4	30	30	35
	WHOA-WHA	13	22	6.9	10	25	35

	WOHA	13	56	6.7	40	55	65
3000 Hz	WHA	13	36	5.5	30	35	45
	WHOA-WHA	13	20	6.6	10	20	30

	WOHA	13	56	7.7	45	55	70
4000 Hz	WHA	13	38	6.0	30	35	50
	WHOA-WHA	13	18	7.5	10	20	35

n: Number of subjects; WOHA: Without hearing aids; WHA: With hearing aids.

Table 4 shows the results from the IOI - HA questionnaire, the scores for each question (1 to 7), factor 1, factor 2, total scores, mean values, medians, and standard deviations observed for the 13 included individuals.

**Table 4:** IOI - HA questionnaire results considering the scores for each question (1 to 7), factor 1, factor 2, total score, mean values, median, and standard deviation for the 13 individuals.

Subject	Q1	Q2	Q3	Q4	Q5	Q6	Q7	F1 (1, 2, 4, 7)	F2 (3, 5, 6)	Total score
1	5	5	4	5	4	3	5	20	11	31
2	2	5	5	3	5	5	5	15	15	30
3	5	5	4	4	5	4	5	19	13	32
4	5	5	4	5	3	4	5	20	11	31
5	5	5	4	5	4	4	3	18	12	30
6	3	5	4	3	5	3	5	16	12	28
7	5	3	5	5	4	4	5	18	13	31
8	5	5	4	5	5	4	5	20	13	33
9	5	5	4	5	5	4	5	20	13	33
10	5	5	5	5	5	5	5	20	15	35
11	5	5	5	5	5	5	5	20	15	35
12	5	5	5	5	5	5	5	20	15	35
13	5	5	5	5	5	5	5	20	15	35
Mean	4.5	4.8	4.5	4.6	4.6	4.2	4.8	19	13	32
Median	5	5	4.5	5	5	4	5	20	13	32
SD	0.9	0.5	0.5	0.7	0.6	0.7	0.5	1.8	1.6	2.4

Q: Question; F: Factor; SD: Standard deviation.

## DISCUSSION

Eight of the 13 included subjects were males (Table 1). The literature reports similar gender incidence rates^10^, ^11^, ^12^. The estimated male to female ratio of occurrence of malformations is 2.5:1^13^.

All patients included in this study had bilateral malformation, contrary to what other authors have observed. According to them, unilateral malformations were more prevalent^10^, ^11^, ^13^, ^14^. Our data was in agreement with a study^12^that found similar prevalences for bilateral and unilateral malformations.

Table 1 shows that moderate conductive hearing loss was the most common type of hearing loss found in our patients, followed by severe conductive hearing loss, and severe mixed hearing loss. Authors^15^ have reported that congenital aural atresia causes, at best, moderate conductive hearing loss.

Congenital stenosis or atresia of the ear canal may be partial or complete, and is frequently accompanied by malformations of the pinna, ossicles, middle ear cavity, and otic capsule, resulting in a vast array of types of hearing loss, ranging from moderate conductive to severe mixed hearing loss^16^.

The subjects included in this study claimed to be systematic users of hearing aids (Table 1). Authors^14^ have indicated that in cases of pediatric bilateral congenital aural atresia with preserved cochlear function the patients should be offered bone conduction hearing aids until a decision is made about surgery, before they are sent to school. The authors have noted that hearing aids should be offered as soon as possible to promote early auditory stimulation and the development of speech and language acquisition.

A study carried out with 20 children with ear malformations found that 36% of the subjects with unilateral malformations rarely used bone conduction hearing aids, and that 44% of the children with bilateral malformation always had them on. It is possible that the latter group wore hearing aids more frequently as a result of their bilateral involvement^1^.

Table 2 shows better SRTN at 65 dBA when patients wore bone conduction hearing aids than when they did not wear them.

Signal to noise ratios may be presented in the form of positive or negative numbers, and are expressed in decibels (dB)^17^. When the intensity of speech is higher than the intensity of noise, the SNR is positive, making it easier for the listener to understand what is being said. However, when the intensity of speech is lower than the intensity of noise, speech recognition is impaired. Subjects able to perform well in the presence of negative signal to noise ratios are deemed to have better hearing function.

Many studies have looked into speech recognition in noise tests to compare individuals wearing conventional bone conduction hearing aids and bone-anchored hearing aids. Some authors have reported better results for BAHA users^18^, ^19^, ^20^, ^21^, ^22^, ^23^, ^24^, ^25^, while others failed to observe such difference^26^, ^27^.

A study conducted with 122 subjects on speech recognition in noise at an SNR of + 6 dB found a mean improvement in speech recognition of 35.5% when subjects wore bone conduction hearing aids and a mean improvement of 41.8% when they wore BAHA, when compared to subjects without hearing aids. The 6.3% difference between BAHA and conventional bone conduction hearing aids was statistically significant^25^.

In Brazil, studies on the fitting of hearing aids in individuals with ear malformations are scarce. A study was carried out on two individuals with craniofacial malformations and bilateral moderate conductive hearing loss wearing conventional bone conduction hearing aids and BAHA. The author looked into free field tone thresholds and speech recognition in noise using the staggered spondaic word test, with the subjects not wearing hearing aids, with conventional bone conduction hearing aids on, and with BAHA on, to conclude that both devices yielded satisfactory levels of hearing gain. In regards to speech recognition, the BAHA outperformed the other devices, but failed to show evidences of improvement when compared to monaural and binaural fittings of the two types of hearing aids analyzed^28^.

According to a study carried out at a high complexity care center^1^, bone conduction hearing aids have improved the speech recognition skills of children with bilateral ear malformations.

Table 3 shows a homogeneous distribution of the data, revealing that all patients in the study improved their hearing thresholds at all analyzed frequencies when they were wearing hearing aids.

The IOI - HA is a sensitive tool that can be used to detect individuals with negative experiences while wearing hearing aids^9^.

Table 4 shows positive mean scores, considering that the maximum possible score is five for each question. Consequently, the analyses of factor 1, factor 2, and total score were also positive, and indicated a good level of subjective satisfaction with the hearing aids.

These results have shown that the individuals included in this study were happy with their hearing aids, contrary to what was observed in other papers^21^, ^23^, ^25^, ^26^, ^29^, in which bone conduction hearing aids were linked with discomfort caused by the pressure the headband applied on the mastoid, skin irritation, acoustic feedback, poor acoustic properties because of the attenuation introduced by the skin and soft tissues in the interaction between the transmitter and the bone, and poor compliance for cosmetic reasons.

## CONCLUSION

Patients showed improved functional gain and reported they were happy with their bone conduction hearing aids, thus showing the benefits provided to the studied population by this type of hearing aids.
